# Patients' perspectives and experiences regarding medication reviews: A cross-sectional survey study

**DOI:** 10.1016/j.rcsop.2025.100692

**Published:** 2025-12-05

**Authors:** Charlotte Falke, Fatma Karapinar-Çarkit, Wilma Knol, Marcel Bouvy, Toine Egberts, Thomas Kempen, Marcia Vervloet, Mette Heringa

**Affiliations:** a Division of Pharmacoepidemiology and Clinical Pharmacology, Utrecht Institute for Pharmaceutical Sciences, Faculty of Science, Utrecht University, Utrecht, the Netherlands; b Department of Clinical Pharmacy & Toxicology, Maastricht University Medical Center+, Maastricht, the Netherlands; c Department of Clinical Pharmacy, CARIM Cardiovascular Research Institute Maastricht, Maastricht University, Maastricht, the Netherlands; d Geriatric Medicine Department and Expertise Centre Pharmacotherapy in Old Persons, University Medical Centre Utrecht, Utrecht University, Utrecht, the Netherlands; e Department of Clinical Pharmacy, University Medical Centre Utrecht, Utrecht, the Netherlands; f Department of Pharmacy, Uppsala University, Uppsala, Sweden; g Nivel, Netherlands Institute for Health Services Research, Utrecht, the Netherlands; h SIR Institute for Pharmacy Practice and Policy, Leiden, the Netherlands

**Keywords:** Medication review, Patients' perspectives, Primary care, Polypharmacy

## Abstract

**Background:**

Medication reviews are conducted worldwide to reduce medication-related problems. However, real-world patient perspectives and experiences remain underexplored. This study aimed to explore patients' perspectives and experiences regarding medication reviews and to assess differences across levels of polypharmacy.

**Methods:**

A cross-sectional online survey was conducted among a panel of Dutch pharmacy visitors. Topics included perspectives and experiences regarding medication reviews. Descriptive statistics were used, and Chi-square tests were used to assess differences between levels of polypharmacy (non-polypharmacy: 1–4 medications; polypharmacy: 5–9 medications; hyperpolypharmacy: ≥10 medications).

**Results:**

Among 4395 respondents (median age: 71; 43 % female), 48 % were aware of the existence of medication reviews, and 85 % deemed these important. Patients valued discussing the appropriateness, efficacy, side-effects, and risks of medication more than practical medication-related issues. These perspectives were consistent across polypharmacy levels. Overall, 1176 patients (27 %) had experienced a review (non-polypharmacy: 22 %; polypharmacy: 29 %; hyperpolypharmacy: 39 %). Of these, 92 % felt able to ask questions, and 62 % felt treatment options were sufficiently discussed. Patients reported that their review resulted in better medication understanding (68 %), increased confidence in medication usage (65 %), and fewer health issues (40 %). Patients with non-polypharmacy reported improvements in follow-up agreements (9 %) and involvement of secondary healthcare providers (11 %) less frequently than those with polypharmacy (14 %/15 %) and hyperpolypharmacy (19 %/26 %).

**Conclusion:**

Most patients valued medication reviews, yet only half were aware of their existence. Generally, levels of polypharmacy hardly impacted patient perspectives. Based on patients' experiences, shared decision-making, follow-ups, and multidisciplinary approaches could be better implemented in medication reviews.

## Highlights

• Patients value medication reviews, although only half of them were aware that these services exist.
• Patient perspectives are largely consistent over different levels of polypharmacy.
• Patients prioritize appropriateness, efficacy, side-effects, and risk of medicines over practical use issues.
• Better shared decision-making, follow-up agreements, and a multidisciplinary approach could strengthen medication reviews.
• Hyperpolypharmacy patients more often mentioned follow-up agreements and a multidisciplinary approach as improvements.

## 1 Introduction

As the population ages, multimorbidity increases. This comes with an increase in polypharmacy (chronic use of ≥5 medications) and hyperpolypharmacy (≥10 medications), leading to more medication-related problems.^1^ A clinical medication review is a widely implemented instrument aimed at reducing these medication-related problems. It is defined as a comprehensive evaluation of a patient's medicines in the context of their clinical conditions by a pharmacist and physician, after discussing medication use with the patient.^2^ Several studies have shown that clinical medication reviews help optimize medication use and reduce medication-related problems.^3^, ^4^, ^5^, ^6^, ^7^, ^8^, ^9^ In the Netherlands, medication reviews are included in the multidisciplinary clinical guideline for polypharmacy in older patients. The current guideline states that the general practitioner and the pharmacist should consider a medication review in patients with an expected high risk—particularly those aged ≥75 with hyperpolypharmacy and/or established frailty—or in response to possible medication-related problems. In 2022, over 100,000 medication reviews were conducted among patients with varying levels of polypharmacy.^10^^,^^11^

Despite the growing role of medication reviews in pharmaceutical care, there is limited real-world data on patients' perspectives and experiences with these medication reviews.^12^^,^^13^ This is relevant as positive patient perspectives and experiences are linked to the effectiveness of such reviews.^14^ Existing studies are mostly small-scale interview studies,^14^, ^15^, ^16^, ^17^, ^18^ often limited in generalisability by their clinical settings and a narrow focus on older patients with polypharmacy. Few studies have examined patient recruitment or satisfaction with medication reviews,^19^, ^20^, ^21^, ^22^ revealing both patients' appreciation for the service and their limited awareness of it. No large-scale survey studies focus on perspectives toward and experiences with medication reviews outside of a clinical study setting, using real-world data. Furthermore, prior studies did not differentiate between patients' perspectives across different levels of polypharmacy, even though polypharmacy or hyperpolypharmacy are commonly used to define eligibility for medication reviews in research and clinical practice.^11^^,^^23^ Therefore, this study aimed to explore patients' perspectives and experiences regarding medication reviews and to assess differences across levels of polypharmacy (i.e., non-polypharmacy, polypharmacy, and hyperpolypharmacy).

## 2 Methods

### 2.1 Study setting, design, and population

In 2023, a cross-sectional online survey was conducted among Dutch pharmacy visitors to explore their perspectives on and experiences with medication reviews. All patients on the AMP Research & Consultation in Healthcare Patient panel were approached to participate in this study. This is a representative panel of patients who visit Dutch community pharmacies regularly and have been recruited by their community pharmacy to participate in surveys on medication-related topics. At the time of the survey, the panel consisted of 20,779 individuals; 51 % were male, and 97 % reported chronic use of at least one medication upon panel inclusion.^24^ AMP collaborates with the Utrecht Pharmacy Practice Network for Education and Research (UPPER), Division of Pharmacoepidemiology and Clinical Pharmacology.^25^ The reporting of this study has been guided by the Consensus-Based Checklist for Reporting of Survey Studies (CROSS).^26^

Inclusion criteria for this study were respondents who used at least one long-term (>3 months) prescription medication. Incomplete questionnaires were included, provided that the pivotal mid-questionnaire item on having had a medication review had been answered. Patients who did not respond to this question (question 10, Appendix 1) were excluded because it was unclear whether their answers reflected their experiences and perspectives on medication reviews.

### 2.2 Survey and administration

Patients were invited by a one-time email to participate in the survey in July 2023. The online survey tool used for data collection was Survalyzer. The questionnaire was in Dutch and consisted of 27 questions, including 12 multiple-choice questions, 3 open numerical questions, 1 open-ended question, and 11 questions with a 5-point Likert-scale (see Appendix 1 for the translated questionnaire and Appendix 2, Fig. 1 for the questions used for this study). The questionnaire was divided into three topics: patient characteristics, perspectives regarding communication about medication use, and experiences with medication reviews. As no validated questionnaire was available, questions were based on literature findings and a thorough discussion by the researchers.^8^^,^^16^^,^^17^^,^^27^ Three patient representatives (two of them being older than 60 and chronically using medication) reviewed the questionnaire; based on their input, the wording of several questions was adapted to optimize comprehensibility.

Questions on patient characteristics included socio-demographic questions on gender, age, education level, health literacy, number of long-term (>3 months) prescription medications, and awareness of medication reviews and whether they had a medication review in the past. To assess health literacy skills, the Set of Brief Screening Questions was used.^28^ Questions about the perspectives included the top three healthcare providers that patients preferred to discuss their medication use with, the importance of medication reviews, and topics about medication use that patients felt should be discussed (e.g., efficacy or risks of medication). Questions about the experience with medication reviews were divided into whether the patient had a medication review or not. If not, the reasons for not having had a medication review were asked. Patients who were unsure whether or not they experienced a medication review were not further questioned about their experience. For patients who had a medication review, the topics and process discussed during the review (e.g., follow-up agreements), the outcomes they reported, and the improvements they suggested were examined. Follow-up agreements made at the end of the medication review consultation specify the next steps to be taken after the consultation, including who will carry them out and within what timeframe. A medication review was explained as follows: “Pharmacists and general practitioners sometimes invite (older) people who use multiple medications for a medication review. This thorough conversation covers all the medications a person uses and discusses potential complaints, side effects, and questions. Often, the pharmacist conducts the conversation and then consults the general practitioner and/or medical specialist about any necessary medication adjustments.”

### 2.3 Data analysis

**T**he patients' perspectives and experiences regarding medication reviews were first analysed in general. To evaluate differences in perspectives and experience between levels of polypharmacy, the levels were classified based on the number of long-term prescription medications: non-polypharmacy (1–4 medications), polypharmacy (5–9 medications), and hyperpolypharmacy (≥10 medications).

For the analysis, the questions with 5-point Likert scales were transformed into three categories to show only positive, neutral, and negative responses, as subgroup responses on the original Likert scale could be small per subcategory. Age groups were categorized according to the Dutch guidelines into <65, 65–74, and ≥ 75 years.^11^^,^^23^ The original education level categories of the questionnaire were consolidated from six to three, still following the International Standard Classification of Education (ISCED).^29^ The health literacy level was calculated as a mean score over the three questions, in which the Likert-scale answers were graded 0–4. A mean score ≤ 2 is interpreted as low health literacy.^28^ In the answers regarding the question on improvements in medication reviews, hospital specialists and nurses were aggregated into secondary healthcare professionals. Free text fields were analysed by two researchers, and responses that matched predefined options were aggregated accordingly. The ranking question in which patients selected their top three healthcare providers to discuss their medication use was transformed into a frequency measure, expressed as the percentage of patients who included a healthcare provider in their top three. If questions regarding experience with medication reviews were answered with “I don't remember”, this was considered a missing answer in the analysis.

Descriptive statistics were used to report the patient characteristics and perspectives and experiences of the study population. Levels of polypharmacy were compared for differences in proportions using Chi-square tests. When more than 20 % of the cells had counts <5, Fisher's exact test was used. A sensitivity analysis based on completed cases only was performed. The data were analysed using IBM SPSS Statistics for Windows, Version 29.0 (IBM Corp., Armonk, N.Y., USA).

### 2.4 Ethics and confidentiality

The Institutional Review Board of the Division of Pharmacoepidemiology and Clinical Pharmacology of Utrecht University approved the research protocol, with reference number UPF2310. Patients gave informed consent, and answers were processed anonymously.

## 3 Results

Of the 20,779 AMP panel members, 5286 (25 %) responded to the survey. One patient did not use any long-term medication and was excluded. In addition, 890 patients (17 %) were excluded because they did not fill in the question regarding experiencing a medication review. Therefore, 4395 patients were included in the analyses (Appendix 3, Fig. 2).

The study population had a median age of 71 (IQR, 64–76) years, with 43 % being female, and the median number of long-term medications used was 5 (IQR, 3–7). The non-polypharmacy group consisted of significantly younger patients, more females, and patients with a higher level of education (Table 1). Of the total study population 48 % were aware of the existence of medication reviews. The awareness of medication reviews was lower among non-polypharmacy patients. Of the study population, 27 % stated they had experienced a medication review, 68 % had no experience with medication reviews, and 5 % of patients were uncertain if they had experienced a medication review (Appendix 3, Fig. 3). Non-polypharmacy and polypharmacy patients experienced a medication review less often than hyperpolypharmacy patients (Table 1). Of the hyperpolypharmacy patients 56 % reported no experience with medication reviews.

**Table 1:** Patient characteristics in numbers and percentages, in total (*n* = 4395) and stratified by the levels of polypharmacy. *P*-values for comparisons between levels of polypharmacy per characteristic.

Characteristics	Total (n = 4395)	Non-polypharmacy (*n* = 2087)	Polypharmacy (*n* = 1926)	Hyper-polypharmacy (*n* = 382)	p-value
**Median number of medications (IQR)**	**5 (3–7)**	**3 (2–4)**	**6 (5–7)**	**11 (10−13)**	
Age group					**< 0.001**
25–64	1184 (27 %)	677 (32 %)	422 (22 %)	85 (22 %)	
65–74	1829 (42 %)	831 (40 %)	830 (43 %)	168 (44 %)	
75–94	1382 (31 %)	579 (28 %)	674 (35 %)	129 (34 %)	
Gender⁎					**< 0.001**
Female	1880 (43 %)	999 (48 %)	735 (38 %)	146 (38 %)	
Education level⁎					**< 0.001**
Low	792 (19 %)	333 (17 %)	368 (20 %)	91 (25 %)	
Medium	1586 (38 %)	725 (36 %)	722 (39 %)	139 (39 %)	
High	1802 (43 %)	929 (47 %)	743 (41 %)	130 (36 %)	
Health literacy⁎					0.105
Low	123 (3 %)	53 (3 %)	53 (3 %)	17 (5 %)	
Awareness of the existence of medication reviews					**0.007**
Yes	2113 (48 %)	985 (47 %)	915 (48 %)	213 (56 %)	
Medication Review experience					**< 0.001**
Yes	1176 (27 %)	465 (22 %)	563 (29 %)	148 (39 %)	
No	3006 (68 %)	1533 (74 %)	1260 (65 %)	213 (56 %)	
Unsure	213 (5 %)	89 (4 %)	103 (5 %)	21 (5 %)	

⁎ Missing: gender 7 (0.2 %), education level 215 (4.9 %), health literacy 175 (4.0 %).

Of the patients who had experienced a medication review, 50 % had their medication review with a pharmacist, 41 % with a general practitioner, and 9 % with a medical specialist (Table 6, Appendix 4). Among the non-polypharmacy patients, there was a higher rate of patients who had their medication reviewed with the general practitioner than with a pharmacist (50 % vs 41 %, p 〈0,001).

Most patients would prefer to discuss medication use with their general practitioner, followed by the pharmacist and the medical specialist (Table 2). Nurses were mentioned least by patients. Differences emerge in the rankings of healthcare professionals based on the level of polypharmacy. Among non-polypharmacy patients, pharmacists are ranked second (68 %) and medical specialists third (55 %). However, as medication use increases, this order shifts: among hyperpolypharmacy patients, medical specialists move to second place (80 %) while pharmacists drop to third (64 %).

**Table 2:** Role of healthcare providers in medication use guidance, in total (*n* = 4395) and stratified by the levels of polypharmacy.

	The most frequently listed healthcare providers in the top 3 preferred by the patient to have a conversation with about medication use							
Medication	General practitioner	Pharmacist	Medical specialist	General practice nurse or assistant	Pharmacy technician	Nurse	Geriatric nurse or specialist	Community or homecare nurse
Total	95 %	67 %	62 %	43 %	20 %	6 %	3 %	1 %
Non-polypharmacy	96 %	68 %	55 %	45 %	25 %	5 %	2 %	1 %
Polypharmacy	94 %	65 %	66 %	43 %	17 %	7 %	3 %	1 %
Hyperpolypharmacy	94 %	64 %	80 %	34 %	13 %	9 %	3 %	1 %

- **General practitioners** are primary care physicians who provide first-line medical care

- **Pharmacists** are medication experts responsible for dispensing medicines, reviewing pharmacotherapy, and providing counselling;

- **Medical specialists** refer to hospital-based physicians such as cardiologists, internists, or neurologists

- **General practice nurses or assistants** support general practitioners in delivering routine primary care and chronic disease management

- **Pharmacy technicians** assist pharmacists with dispensing and patient communication;

- **Nurses** provide general nursing care, predominantly in clinical settings

- **Geriatric nurses or specialists** focus on the care of older adults with complex health needs i**n clinical settings**;

- **Community or homecare nurses** provide nursing care and support in patients' homes.

As shown in Table 3, 3745 patients (85 %) considered a medication review important, with no differences between patients with and without (hyper)polypharmacy. Most topics that could be discussed during a medication review were regarded as important by patients, i.e., the appropriateness of medication (90 %), the efficacy of medication (89 %), side-effects (86 %), and risk of medication (85 %). Patients regarded concerns regarding medication (68 %) and practical problems with using medication (51 %) as less important to discuss. The importance of discussing practical problems differed significantly between patients with non-polypharmacy (52 %) and polypharmacy (49 %) compared to those with hyperpolypharmacy (58 %).

**Table 3:** Numbers and percentages of patients indicating medication review and certain discussion topics about medication use as important, in total (*n* = 4395) and stratified by levels of polypharmacy. *P*-values for comparisons between levels of polypharmacy.

	Total (n = 4395)	Non-polypharmacy (*n* = 2087)	Polypharmacy (*n* = 1926)	Hyper- polypharmacy (*n* = 382)	p-value
Importance of medication review					
Medication review	3745/4395 (85 %)	1777/2087 (85 %)	1645/1926 (85 %)	323/382 (85 %)	0.208
Importance of topics about medication use					
Appropriateness of medication	3810/4246 (90 %)	1810/1996 (91 %)	1664/1876 (89 %)	336/374 (90 %)	0.338
Efficacy of medication	3829/4293 (89 %)	1814/2021 (90 %)	1677/1894 (89 %)	338/378 (89 %)	0.801
Side-effects	3404/3972 (86 %)	1580/1850 (85 %)	1519/1769 (86 %)	305/353 (86 %)	0.932
Risks of medication	3589/4239 (85 %)	1689/1987 (85 %)	1573/1877 (84 %)	327/375 (87 %)	0.226
Patients' concerns regarding medication	2592/3802 (68 %)	1185/1753 (68 %)	1163/1707 (68 %)	244/342 (71 %)	0.604
Practical problems with using medication	**1891/3705 (51 %)**	**879/1701 (52 %)**	**820/1670 (49 %)**	**192/334 (58 %)**	**0.031**

In total, 1176 (27 %) patients had experience with a medication review. Patients who did not have a medication review were asked for the reason (Appendix 5, Fig. 3). In general, “I did not receive an invitation” was the most frequent reason (54 %), followed by unawareness of the existence of a medication review (43 %) and absence of medication-related problems (39 %). Four reasons for not having had a medication review significantly differed between patients across different levels of polypharmacy, namely not receiving an invitation, not qualifying for a medication review, no problems with taking the medication, and not experiencing side-effects. Non-polypharmacy patients more often stated they had no side-effects (28 %), and thus did not need a medication review, than polypharmacy (21 %) and hyperpolypharmacy patients (12 %) (*p* < 0.001). Non-polypharmacy patients also more often stated they had no problems taking their medication (45 %) than polypharmacy (34 %) and hyperpolypharmacy (24 %) patients (p < 0.001). The same applied to not receiving an invitation or not qualifying for a medication review, which was reported by 48 % and 4 % of non-polypharmacy patients, compared to 61 % and 1 % of both polypharmacy and hyperpolypharmacy patients, respectively (p < 0.001 for both).

In the sensitivity analysis, the same distributional patterns were observed; however, due to the smaller sample sizes, the statistical significance was slightly reduced (Appendix 4 Table 7).

Table 4 details the topics that were discussed during the medication review and the outcomes of the review according to patients who had a medication review. The most discussed topics were: the opportunity to discuss questions (92 %), the aim of the conversation (87 %), health issues (83 %), and priorities in these issues (77 %). Less often discussed topics were follow-up agreements (76 %) and the different treatment options (62 %). When comparing the patients with different levels of polypharmacy, there was a significant difference in the discussion of the follow-up agreements after the medication review; this was discussed with 78 % of the non-polypharmacy, 76 % of the polypharmacy, and 65 % of the hyperpolypharmacy patients (*p* = 0.009).

**Table 4:** Numbers and percentages of topics discussed during a medication review and outcomes of the medication review, in total and stratified by the levels of polypharmacy. *P*-values for comparisons between levels of polypharmacy.

Topics discussed during medication review	Total	Non-polypharmacy	Polypharmacy	Hyper-polypharmacy	
	Agree	Agree	Agree	Agree	p-value
I could ask all my questions	1025/1113 (92 %)	398/434 (92 %)	493/538 (92 %)	134/141 (95 %)	0.197⁎
Aim conversation was clear	968/1108 (87 %)	370/426 (87 %)	474/541 (88 %)	124/141 (88 %)	0.831⁎
Health issues were discussed	903/1085 (83 %)	362/418 (87 %)	429/529 (81 %)	112/138 (81 %)	0.138
Priorities in issues	816/1060 (77 %)	325/415 (78 %)	389/510 (76 %)	102/135 (76 %)	0.564
Follow-up agreements	**797/1055 (76 %)**	**322/412 (78 %)**	**385/505 (76 %)**	**90/138 (65 %)**	**0.009**
Different treatment options	633/1024 (62 %)	258/391 (66 %)	297/503 (59 %)	78/130 (60 %)	0.185
Outcomes of the medication review					
Better understanding	677/1002 (68 %)	266/373 (71 %)	330/498 (66 %)	81/131 (62 %)	0.278
More faith medication	650/1006 (65 %)	255/377 (68 %)	314/499 (63 %)	81/130 (62 %)	0.614
Alteration in medication	561/1074 (52 %)	222/430 (52 %)	273/514 (53 %)	66/130 (51 %)	0.846
Medication use improved	463/937 (49 %)	173/335 (52 %)	233/474 (49 %)	57/128 (45 %)	0.107
Fewer health issues	**383/958 (40 %)**	**176/360 (49 %)**	**171/476 (36 %)**	**36/122 (30 %)**	**<0.001**

⁎ Fisher's exact test.

Patients reported different outcomes due to the medication review. Most often, better understanding (68 %) and more faith in medication were selected (65 %). Half of the patients had experienced an alteration in medication and reported that their medication use improved. Forty percent of patients reported fewer health issues. Only the experience of fewer health issues differed significantly between patients with non-polypharmacy (49 %), polypharmacy (36 %), and hyperpolypharmacy (30 %; Table 4).

In the sensitivity analysis, the same distributional patterns were observed; however, due to the smaller sample sizes, the statistical significance was slightly reduced (Appendix 5 Table 8).

Table 5 lists the improvements patients reported for medication reviews. Almost half of the patients stated that no improvements were necessary. In total, 52 % of patients had one or more improvements: more attention to personal wishes (25 %), more involvement of the general practitioner (23 %) or secondary healthcare professionals (15 %), a costless medication review (13 %), and/or better communication about follow-up agreements (13 %).

**Table 5:** Improvements for future medication reviews stratified by the levels of polypharmacy. *P*-values for comparisons between levels of polypharmacy.

	Total⁎ (*n* = 1143)	Non-polypharmacy (n = 448)	Polypharmacy (*n* = 550)	Hyperpolypharmacy (*n* = 145)	p-value
Nothing	**549 (48 %)**	**236 (53 %)**	**245 (45 %)**	**68 (47 %)**	**0.036**
More attention to personal wishes	**283 (25 %)**	**92 (21 %)**	**153 (28 %)**	**38 (26 %)**	**0.027**
More involvement of the general practitioner	265 (23 %)	93 (21 %)	132 (24 %)	40 (28 %)	0.196
More involvement of secondary healthcare professionals⁎⁎	**170 (15 %)**	**51 (11 %)**	**82 (15 %)**	**37 (26 %)**	**<0.001**
A costless medication review	147 (13 %)	66 (15 %)	65 (12 %)	16 (11 %)	0.307
Better follow-up agreements	**145 (13 %)**	**42 (9 %)**	**76 (14 %)**	**27 (19 %)**	**0.008**
Other	60 (5 %)	20 (5 %)	31 (6 %)	9 (6 %)	0.610

⁎ Missing: 33 patients.

⁎⁎ Secondary healthcare professionals are an aggregation of hospital specialists and hospital nurses.

Three improvements were significantly different between the patients with varying levels of polypharmacy, namely the attention to personal wishes, the involvement of secondary healthcare professionals, and better follow-up agreements. The involvement of secondary healthcare professionals was mentioned less often by non-polypharmacy patients (11 %) compared to patients with polypharmacy (15 %) and non-hyperpolypharmacy (26 %, *p* < 0.001). Also, better follow-up agreements were less often mentioned by non-polypharmacy patients (9 %, polypharmacy: 14 %; hyperpolypharmacy: 19 %, *p* = 0.008).

## 4 Discussion

This study explored the real-world perspectives and experiences regarding medication reviews of patients with varying levels of polypharmacy and found that only 48 % of patients were aware of the existence of medication reviews, and 27 % actually experienced them. The majority of patients (85 %) found medication reviews important. General practitioners, pharmacists, and medical specialists were considered the preferred healthcare providers to discuss medication use with. Patients attached greater importance to discussing medication appropriateness, efficacy, side-effects, and risks of medication than to practical problems with using medication. Some differences in perspectives were observed across varying polypharmacy levels; however, these differences were minor and may lack clinical significance.

The most common reasons for not having had a medication review were a lack of invitation and patients' unawareness of the availability of medication reviews. Almost all patients who had a medication review felt able to ask questions, and 62 % felt treatment options were adequately discussed. Non-polypharmacy patients more often stated that follow-up agreements were discussed than hyperpolypharmacy patients. Patients reported improved medication understanding, confidence in medication usage, and fewer health issues as outcomes of the medication reviews. Non-polypharmacy patients more often stated they had fewer health issues as a result of the medication review than hyperpolypharmacy patients. Nearly half of the patients who had a medication review reported areas for improvement, most commonly attention to their personal wishes and increased involvement of their healthcare professional. Compared to non-polypharmacy patients, hyperpolypharmacy patients were twice as likely to mention follow-up agreements and more involvement of secondary healthcare professionals as potential improvements.

### 4.1 Comparison to previous literature

Previous studies indicated that older patients with poorer health or complex regimens were more likely to request medication reviews.^21^ Although there is no information about complexity or health status in the questionnaire used in this study, the number of medications is an indication of complexity and multimorbidity; therefore, there was an expected higher prevalence of medication reviews among hyperpolypharmacy patients. Especially since medication reviews are recommended for hyperpolypharmacy patients, older than 75, or polypharmacy patients with frailty in the current Dutch guidelines^11^^,^^23^ and Dutch reimbursement data indicate a higher prevalence.^10^ Medication reviews may sometimes be conducted by community pharmacists with general practitioners, without or with limited involvement of the patient, so it is possible that the actual medication review rates were higher, but the patients were unaware. Additionally, 41 % of patients reported that their medication review was conducted by their general practitioner, which may suggest it was a different type of medication consultation than a clinical medication review with clear pharmacist involvement. In this study, a low awareness was found of the existence of medication reviews. This is in line with previous studies, where general awareness about medication reviews by pharmacists was low.^20^^,^^22^

With regards to the perspectives, patients are less likely to discuss medication use with pharmacists than with general practitioners, and, in the case of hyperpolypharmacy patients, with general practitioners and medical specialists. Previous research acknowledges a lack of understanding of the role of pharmacists in consultation about medication use among patients with chronic diseases, although the high accessibility of pharmacists for patients is mentioned.^30^, ^31^, ^32^ Medication reviews were generally considered important by patients in this study, which is consistent with previous findings.^14^, ^15^, ^16^^,^^18^^,^^19^^,^^33^ As patients get older, they are at a higher risk of medication-related problems due to their higher risk of polypharmacy and an increased susceptibility due to changes in pharmacokinetics and pharmacodynamics.^34^ It seems patients are aware of these medication-related risks and the importance of medication reviews. Previous research on patients´ perspectives on medication management found that older patients have built-in routines, more acceptance of their situation, and lower medication knowledge.^35^^,^^36^ This might explain why patients find appropriateness and efficacy of their medication more important than practical issues, especially since older patients formed the majority of the respondents. Practical problems were considered slightly more important by hyperpolypharmacy patients. This difference was to be expected, as hyperpolypharmacy patients have a higher risk of medication use complexity and therefore, might find practical problems more important.^37^

Patients who experienced a medication review indicated that many topics were discussed during their review, including the aim of the review, health issues, and questions. Previous studies emphasized the importance of these topics in the process and content of a review.^14^, ^15^, ^16^, ^17^, ^18^ This study shows that these topics are well integrated in real-life medication reviews. Yet only 62 % of the patients reported that different treatment options were discussed with them. While a single clinically appropriate treatment option may have been available, this finding could also reflect insufficient patient involvement in the decision-making process. Previous research has highlighted the importance of shared decision-making in clinical care.^38^, ^39^, ^40^ Providing information about the risks associated with specific medications, along with a clear explanation of alternative treatment options, has been shown to support this process effectively.^38^^,^^39^

According to the patients in this study, outcomes of a medication review were improved medication understanding, confidence in medication usage, and fewer health issues. This is similar to studies in clinical settings, with medication reviews resulting in fewer medication-related problems.^9^ In this study, non-polypharmacy patients more often reported fewer health issues as an outcome of the review than polypharmacy and hyperpolypharmacy patients. Hyperpolypharmacy and polypharmacy patients likely have a poorer overall well-being than non-polypharmacy patients, which may contribute to their decreased sense of improvement in health issues.^1^^,^^21^ Patients' appreciation of medication reviews in this study appeared to be positive, as 48 % of those who had a review did not suggest any improvements, indicating they were likely satisfied with the review. Suggested improvements were greater attention to personal wishes and increased involvement of secondary healthcare providers. These suggestions are in line with previous literature,^40^ which calls for enhanced implementation of shared decision-making. A multidisciplinary approach has also been recommended in studies on in-hospital medication reviews among older patients with polypharmacy.^14^^,^^16^^,^^40^ Although medication reviews may have involved multiple healthcare professionals, patients may have perceived only the involvement of the professional they directly consulted, typically the pharmacist or general practitioner. There was a difference in suggested improvements between non-polypharmacy patients and hyperpolypharmacy patients, with the hyperpolypharmacy patients wanting more involvement of secondary healthcare professionals and better follow-up agreements. Hyperpolypharmacy patients are more likely to have multimorbidity, be under the care of multiple healthcare providers, and receive health recommendations from all of them, which can lead to confusion.

### 4.2 Strengths/limitations

This study included real-world perspectives and experiences of patients, with a large, diverse sample across different age groups and levels of polypharmacy. This reflected the real-world opinion of patients on medication reviews and allowed differentiation between different patient groups.

There are also some limitations. First, certain patients, including those with only primary education, low health literacy, and women, were underrepresented, potentially skewing the generalizability. As the missing values on gender, education level, and health literacy were relatively low and evenly distributed across the levels of polypharmacy, their impact on the generalisability of the findings is expected to be limited. Second, a potential misinterpretation of the term “medication review” may have led to more patients stating they had a medication review, as partial medication consultations by physicians may have been misclassified as full reviews. Data on actual medication reviews performed with the respondents were unavailable; therefore, the answers given cannot be verified. Third, the patient-reported number of medications used could be different from the number used according to healthcare providers, and patients may include or exclude non-prescription drugs. This could impact the classification of the medication use level. On the other hand, the patient-reported number of medications used may be closer to reality than pharmacy/healthcare records. And last, there could be recall bias based on the topic of the survey, as some respondents may have experienced a medication review more recently than others.

### 4.3 Clinical implications

This study highlights the need to raise awareness of medication reviews, as patients consider them important, yet not all are aware of their existence. Especially since medication reviews could potentially improve medication understanding, confidence in medication usage, and reduce health issues.^9^ This study also indicates that awareness of the role of the pharmacist in consultations on medication use can be improved. The overall experiences are good, but better implementation of shared decision-making with more attention to personal wishes and follow-up agreements, and a multidisciplinary approach (including initial prescribers) could further improve medication reviews to meet patients' needs, especially for hyperpolypharmacy patients. More involvement of secondary healthcare providers is important and could be supported by clear communication channels and collaboration platforms.^41^

To further strengthen patient engagement in medication reviews, future research could examine how patients themselves envision an optimal review process, including the desired roles of different health care professionals and the integration of shared decision-making. It would also be valuable to investigate whether such preferences vary across patient groups. In addition, future research could include surveys administered by healthcare professionals as part of the review or an online survey shortly after medication reviews to capture real-time experiences outside a research setting. Linking these patient experiences and patient-reported outcomes to the perspectives of health care providers and to clinical outcomes will provide a more comprehensive understanding of how medication reviews can be optimized in routine practice.

## 5 Conclusion

Most patients found medication reviews important, yet awareness of their existence remains low. Overall, patient perspectives are consistent over different levels of polypharmacy. However, in experiences, there are some different priorities for the content and process of reviews. The overall experiences are good, but better awareness, better implementation of shared decision-making and discussing follow-up agreements, and multidisciplinary approaches could further improve medication reviews to meet patients' needs and perspectives.

## CRediT authorship contribution statement

**Charlotte Falke:** Writing – review & editing, Writing – original draft, Visualization, Software, Methodology, Investigation, Formal analysis. **Fatma Karapinar-Çarkit:** Writing – review & editing, Supervision, Methodology, Formal analysis. **Wilma Knol:** Writing – review & editing, Supervision, Methodology. **Marcel Bouvy:** Writing – review & editing, Supervision, Methodology, Funding acquisition, Conceptualization. **Toine Egberts:** Writing – review & editing, Supervision, Methodology. **Thomas Kempen:** Writing – review & editing, Methodology. **Marcia Vervloet:** Writing – review & editing, Methodology, Funding acquisition, Conceptualization. **Mette Heringa:** Writing – review & editing, Supervision, Project administration, Methodology, Funding acquisition, Formal analysis, Data curation, Conceptualization.

## Declaration of generative AI and AI-assisted technologies in the writing process

During the preparation of this work, the author(s) used ChatGPT to refine the wording of the abstract and to improve clarity in selected sections of the text. After using this tool, the author(s) reviewed and edited the content as needed and take(s) full responsibility for the content of the published article.

## Declaration of competing interest

On behalf of all authors, the corresponding author states that there is no conflict of interest.

The survey used in this study was funded by the Dutch Ministry of Health, Welfare and Sport (reference: 201865006.028.080) and was part of a larger project for which this funding was provided.

## Data Availability

Data for this study will be made available to others in the scientific community upon request after publication.

## Appendix A Supplementary data

Supplementary material: Supplementary data Image 1

## References

[1] WHO (2019). https://www.who.int/docs/default-source/patient-safety/who-uhc-sds-2019-11-eng.pdf.

[2] Zermansky A., Petty D., Raynor D., Lowe C., Freemantle N., Vail A. (2002). Clinical medication review by a pharmacist of patients on repeat prescriptions in general practice: a randomised controlled trial. Health Technol Assess (Rockv).

[3] Verdoorn S., Kwint H.F., Blom J.W., Gussekloo J., Bouvy M.L. (2019). Effects of a clinical medication review focused on personal goals, quality of life, and health problems in older persons with polypharmacy: a randomised controlled trial (DREAMER-study). PLoS Med.

[4] Basheti I.A., Al-Qudah R.A., Obeidat N.M., Bulatova N.R. (2016). Home medication management review in outpatients with chronic diseases in Jordan: a randomized control trial. Int J Clin Pharmacol.

[5] Bryant L.J.M., Coster G., Gamble G.D., McCormick R.N. (2011). The general practitioner-pharmacist collaboration (GPPC) study: a randomised controlled trial of clinical medication reviews in community pharmacy. Int J Pharm Pract.

[6] Fletcher J., Hogg W., Farrell B. (2012). Effect of nurse practitioner and pharmacist counseling on inappropriate medication use in family practice. Can Fam Physician.

[7] Sloeserwij V.M., Hazen A.C.M., Zwart D.L.M. (2019). Effects of non-dispensing pharmacists integrated in general practice on medication-related hospitalisations. Br J Clin Pharmacol.

[8] McCarthy C., Clyne B., Boland F. (2022). GP-delivered medication review of polypharmacy, deprescribing, and patient priorities in older people with multimorbidity in Irish primary care (SPPiRE study): a cluster randomised controlled trial. PLoS Med.

[9] Huiskes V.J.B., Burger D.M., Van Den Ende C.H.M., Van Den Bemt B.J.F. (2017). Effectiveness of medication review: a systematic review and meta-analysis of randomized controlled trials. BMC Fam Pract.

[10] (2025). Optimaliseren van medicatie-gebruik bij ouderen Een evaluatie in opdracht van het ministerie van VWS.

[11] van Marum R.J., Verduijn M.M., Burgers J.S. (2019).

[12] Brandt J., Lê M.-L., Jantscher S. (2020). Medication review service implementation in community pharmacy settings: scoping review with focus on implementation studies. Res Social Adm Pharm.

[13] Michel D.E., Tonna A.P., Dartsch D.C., Weidmann A.E. (2022). Experiences of key stakeholders with the implementation of medication reviews in community pharmacies: a systematic review using the consolidated framework for implementation research (CFIR). Res Social Adm Pharm.

[14] Thevelin S., Pétein C., Metry B. (2022). Experience of hospital-initiated medication changes in older people with multimorbidity: a multicentre mixed-methods study embedded in the OPtimising thERapy to prevent avoidable hospital admissions in multimorbid older people (OPERAM) trial. BMJ Qual Saf.

[15] Baas G., Crutzen S., Smits S., Denig P., Taxis K., Heringa M. (2023). Process evaluation of a pharmacist-led intervention aimed at deprescribing and appropriate use of cardiometabolic medication among adult people with type 2 diabetes. Basic Clin Pharmacol Toxicol.

[16] Kempen T.G.H., Kälvemark A., Gillespie U., Stewart D. (2020). Comprehensive medication reviews by ward-based pharmacists in Swedish hospitals: what does the patient have to say?. J Eval Clin Pract.

[17] McCahon D., Duncan P., Payne R., Horwood J. (2022). Patient perceptions and experiences of medication review: qualitative study in general practice. BMC Primary Care.

[18] Robberechts A., Van Loon L., Steurbaut S., De Meyer G.R.Y., De Loof H. (2023). Patient experiences and opinions on medication review: a qualitative study. Int J Clin Pharmacol.

[19] Cardosi L., Hohmeier K.C., Fisher C., Wasson M. (2018). Patient satisfaction with a comprehensive medication review provided by a community pharmacist. J Pharm Technol.

[20] Zhang Y., Doucette W.R. (2019). Consumer decision making for using comprehensive medication review services. J Am Pharm Assoc.

[21] Farris K.B., Salgado T.M., Aneese N. (2016).

[22] Vogt C.J., Wiegand A., Trajkov K. (2025). Attitudes and expectations toward medication review among community pharmacy customers in Germany: a cross-sectional study. J Pharm Policy Pract.

[23] van Marum R.J., MM M. Verduijn, de Vries-Moeselaar A.C. (2012). Nederlands Huisartsen Genootschap.

[24] Onderzoek A.M.P., in de zorg Advies http://www.ampnet.nl.

[25] Koster E.S., Blom L., Philbert D., Wi Rump, Bouvy M.L. (2014). The Utrecht pharmacy practice network for education and research: a network of community and hospital pharmacies in the Netherlands. Int. J Clin Pharm Ther.

[26] Sharma A., Minh Duc N.T., Luu Lam Thang T. (2021). A consensus-based checklist for reporting of survey studies (CROSS). J Gen Intern Med.

[27] Kempen T.G.H., Van De Steeg-Van Gompel C.H.P.A., Hoogland P., Liu Y., Bouvy M.L. (2014). Large scale implementation of clinical medication reviews in Dutch community pharmacies: drug-related problems and interventions. Int J Clin Pharmacol.

[28] Chew L.D., Bradley K.A., Boyko E.J. (2004). Brief questions to identify patients with inadequate health literacy. Fam Med.

[29] (2025). International Standard Classification of Education Fields of education and training 2013 (ISCED-F 2013)-Detailed field descriptions.

[30] Butler L., Zona S., Patel A.A., Brittle C., Shea L. (2023). How can pharmacists better support patients with chronic diseases? The patient perspective. J Am Pharm Assoc.

[31] Mossialos E., Courtin E., Naci H. (2015). From “retailers” to health care providers: transforming the role of community pharmacists in chronic disease management. Health Pol (New York).

[32] van de Pol J.M., van Dijk L., Koster E.S., de Jong J., Bouvy M.L. (2021). How does the general public balance convenience and cognitive pharmaceutical services in community pharmacy practice. Res Social Adm Pharm.

[33] Yoo A., Fennelly J.E., Renauer M.M. (2022). Comprehensive medication review service by embedded pharmacists in primary care: innovations and impact. J Am Pharm Assoc.

[34] Patel C.H., Zimmerman K.M., Fonda J.R., Linsky A. (2016). Medication complexity, medication number, and their relationships to medication discrepancies. Ann Pharmacother.

[35] Krause O., Ziemann C.T., Schulze Westhoff M. (2023). What do older patients know about their medication? A cross-sectional, interview-based pilot study. Eur J Clin Pharmacol.

[36] Notenboom K., Beers E., Van Riet-Nales D.A. (2014). Practical problems with medication use that older people experience: a qualitative study. J Am Geriatr Soc.

[37] Wimmer B.C., Bell J.S., Fastbom J., Wiese M.D., Johnell K. (2016). Medication regimen complexity and number of medications as factors associated with unplanned hospitalizations in older people: a population-based cohort study. J Gerontol A Biol Sci Med Sci.

[38] Rozsnyai Z., Jungo K.T., Reeve E. (2020). What do older adults with multimorbidity and polypharmacy think about deprescribing? The LESS study - a primary care-based survey. BMC Geriatr.

[39] Moen J., Bohm A., Tillenius T., Antonov K., Nilsson J.L.G., Ring L. (2009). “I don’t know how many of these [medicines] are necessary.”-A focus group study among elderly users of multiple medicines. Patient Educ Couns.

[40] Engelen E.M.C., Knol W., Wuyts S.C.M. (2025). Older patients’ experiences and needs regarding pharmacotherapeutic care in-hospital and after discharge. Patient Educ Couns.

[41] Daliri S., Hugtenburg J.G., ter Riet G. (2019). The effect of a pharmacy-led transitional care program on medication-related problems post-discharge: a before—after prospective study. PloS One.

