# Supplementary material for: Patients' perspectives and experiences regarding medication reviews: A cross-sectional survey study
Source: Explor Res Clin Soc Pharm. 2025 Dec 5;21:100692. doi: 10.1016/j.rcsop.2025.100692 (PMC12765331; doi:10.1016/j.rcsop.2025.100692)
Supplement: Supplementary file 1 — Supplementary material: Supplementary data [file mmc1.docx]

# Appendixes

## Appendix 1:The (translated) questionnaire

1. What is your age? ______________________________ (validation: number)

2. What is your gender?

- Male
- Female
- Other
- Prefer not to say

3. Are you currently taking any long-term prescription medication (more than three months)?

- Yes
- No

4. How many different long-term prescription medications are you taking? ______________________________ (validation: number)

A discussion about your medications is directly related to your health. It is therefore important that the healthcare professionals you discuss this with have sufficient knowledge about your illness and your medications.

5. Which of the following healthcare professionals do you find most suitable to have a conversation with about your medication use? Please select a top 3.

Click on the 3 healthcare professionals that form your top 3. These will appear at the top of the green box. You can adjust the order afterward by dragging the professionals in the green box higher or lower. You can remove a professional from the top 3 in the green box by clicking on the “=” symbol.

- GP
- Practice nurse / GP nurse
- Pharmacist
- Pharmacy assistant
- Medical specialist (in hospital)
- Specialist in elderly care nursing
- Nurse (in hospital)
- Home care/district nursing
- Other (you can specify this in question 5)
- Other healthcare professional (please specify under question 5):

6. How important do you find it to discuss the following topics with a healthcare professional?

Very unimportant | Unimportant | Neutral | Important | Very important | Not applicable

- Side effects I experience
- Risks of my medication
- Practical problems with using my medication (e.g., breaking tablets or administering eye drops)
- Whether my medication is working well
- Whether all my medications are still appropriate
- Concerns I have about my medication

7. There are many people involved in your medication use. To what extent do you think the following people are responsible for ensuring that your medication use is safe and appropriate?

Not at all | Slightly | Partially | Largely | Fully | Not applicable

- Yourself
- GP
- Pharmacist
- Medical specialist in the hospital

Pharmacists and general practitioners sometimes invite (older) people who use multiple medications for a medication review. This thorough conversation covers all the medications a person uses and discusses potential complaints, side effects, and questions. Often, the pharmacist conducts the conversation and then consults the general practitioner and/or medical specialist about any necessary medication adjustments.

8. Were you aware that pharmacists collaborate with GPs to carry out these medication reviews?

- Yes
- No

9. How important do you find it that pharmacists and doctors conduct medication reviews?

- Very unimportant
- Unimportant
- Neutral
- Important
- Very important

10. Have you ever had such a medication review or medication consultation where all your medications were discussed?

- Yes
- No
- I don't remember

11. You indicated that you have had a medication review. Who conducted the conversation with you? (Multiple answers possible)

- Pharmacist
- Another pharmacy staff member/pharmacy assistant
- GP
- Practice nurse / GP nurse
- A hospital healthcare professional
- Other, namely: ______________________________
- I don’t recall

12. How did the conversation go?

Strongly disagree | Disagree | Neutral | Agree | Strongly agree | I don’t remember

- The healthcare professional clearly explained the AIM of the conversation
- I was able to ask all my questions
- The healthcare professional asked about my health issues
- The healthcare professional asked what I found the priority in issues
- The healthcare professional presented different treatment options
- The follow-up after the conversation was agreed upon

13. Were your medications altered as a result of the medication review?

- Yes
- No
- I don’t remember

14. What were the outcomes of the medication review for you?

Strongly disagree | Disagree | Neutral | Agree | Strongly agree | Not applicable

- I now better understand why I take my medication
- I have more faith in my medication
- I experience fewer health issues
- I can use my medication more effectively

15. What would you like to see different in a future medication review? (Multiple answers possible)

- More attention to my personal preferences regarding medication adjustments
- More involvement of the GP
- More involvement of the hospital specialists
- More involvement of the hospital nurse
- That the conversation is costless
- Better follow-up agreements after the conversation
- Other, namely: ______________________________
- Nothing

16. What are the reasons you have not had a medication review? (Multiple answers possible)

- I was not aware this existed
- I prefer to discuss my medication only with my doctor
- I do not qualify for one
- I have not received an invitation from my doctor or pharmacist
- I have no problems with taking my medication
- I do not experience any side effects from my medication
- Due to possible costs
- Other, namely: ______________________________

As people get older, medications sometimes have more disadvantages than benefits. It is therefore good to assess whether they are still necessary. For example, during a medication review or another discussion with your doctor or pharmacist.

17. How important do you find it that healthcare professionals annually assess whether all medications are still necessary for older patients (perform a yearly medication review)?

- Very unimportant
- Unimportant
- Neutral
- Important
- Very important

18. How important do you find the following aspects in a discussion about potentially stopping one of your medications?

Very unimportant | Unimportant | Neutral | Important | Very important | Not applicable

- The healthcare professional asks for my opinion about the medication
- The healthcare professional tells me what is best action for me
- The healthcare professional consults the doctor who initially prescribed the medication
- Explanation of the pros and cons of stopping
- Opportunity to discuss my concerns
- A leaflet or brochure about the pros and cons of stopping
- The possibility to restart the medication if necessary
- The process follows medical guidelines

Certain medications can increase the risk of falls. Healthcare professionals try to consider this by not prescribing these medications to people who are already at high risk of falling or by taking other measures, such as offering physiotherapy.

19. Has a healthcare professional ever discussed the risk of falling due to medications with you?

- No
- Yes, namely: (please specify one or more healthcare professionals): ______________________________

20. How important do you find it that healthcare professionals pay attention to the risk of falling due to medications?

- Very unimportant
- Unimportant
- Neutral
- Important
- Very important

Finally, some questions about your situation and experiences.

21. How often does someone help you read letters or leaflets from your GP, hospital, or other healthcare institutions?

- Never
- Occasionally
- Sometimes
- Often
- Always

22. How confident are you that you can correctly fill in medical forms on your own?

- Never
- Occasionally
- Sometimes
- Often
- Always

**23. How often do you find it difficult to learn more about your health because you do not fully understand written information?**

- Never
- Occasionally
- Sometimes
- Often
- Always

It is important to determine whether the people completing this questionnaire provide a good representation of all residents of the Netherlands. Therefore, we would appreciate it if you could also answer the following questions.

**24. In which province of the Netherlands do you live?**

- Groningen
- Friesland
- Drenthe
- Overijssel
- Flevoland
- Utrecht
- Gelderland
- North Holland
- South Holland
- Zeeland
- North Brabant
- Limburg

**25. What education qualifications have you completed?**
(Multiple answers possible)
Primary education

- VMBO / MAVO / LBO or other lower vocational education
- MBO (MTS, MEAO) or other secondary vocational education
- HAVO / VWO (HBS, MMS)
- HBO / WO or other higher or academic education
- Other, namely: ______________________________

**26. Where were you born?**

- The Netherlands
- Abroad

If the Netherlands: Were both of your parents born in the Netherlands?

- Yes
- No

**27. Do you have any comments regarding this questionnaire?**

## Appendix 2


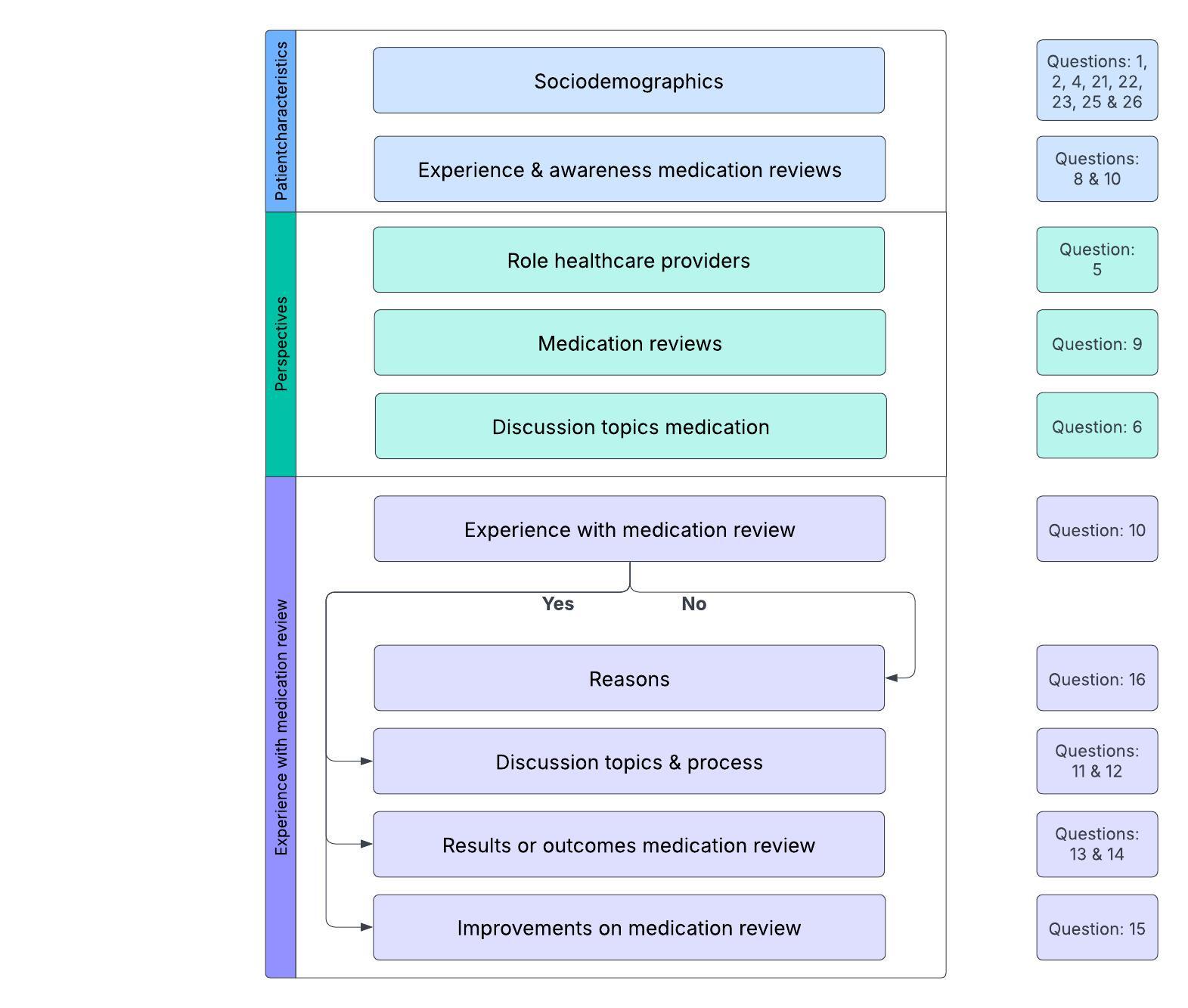


Figure 1: An overview of the questionnaire topics and questions used in this study. All patients were asked questions on patient characteristics and perspectives regarding communication about medication use. Afterward, separate questions were asked to those who experienced a medication review and those who did not. Patients who were unsure whether or not they experienced a medication review were not further questioned about their experience.

## Appendix 3


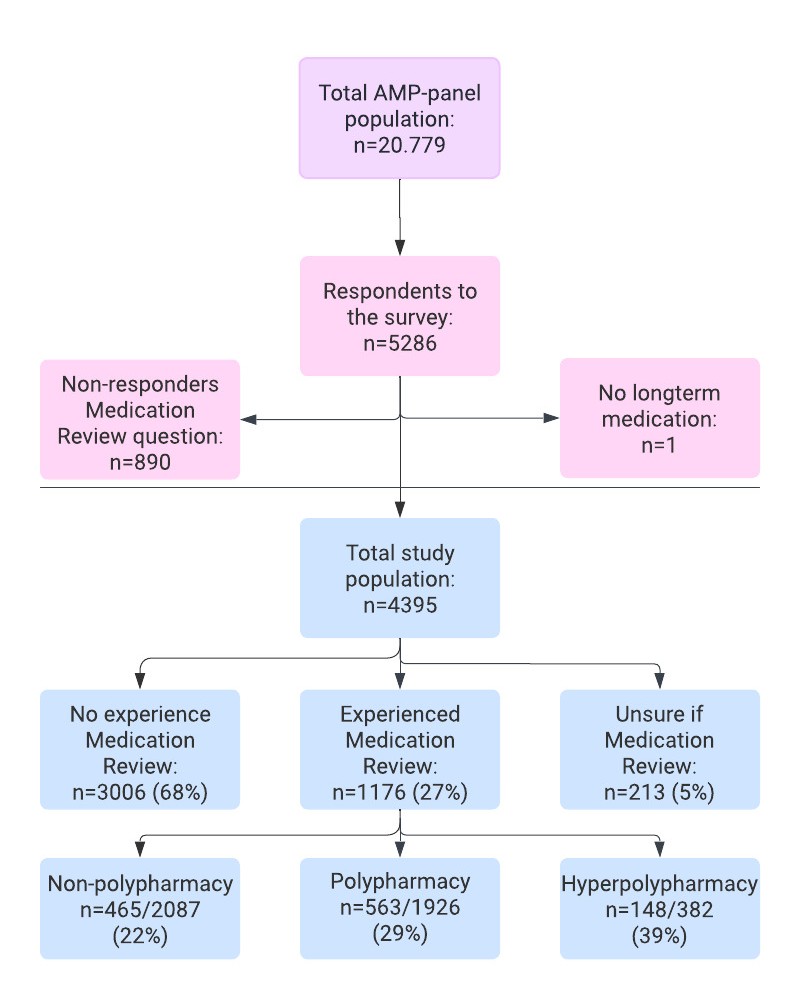


Figure 2: Study population and their experience with a medication review.

## Appendix 4

**Table 6: Healthcare professionals who conducted the medication review, only the most frequent answers are depicted and split into medication groups (n=1176).**

|  | | Total | Non-polypharmacy | Polypharmacy | Hyper-polypharmacy | p-value |
| --- | --- | --- | --- | --- | --- | --- |
| Which healthcare professional conducted the medication review | |  |  |  |  | **<0.001** |
|  | Pharmacy | 568 (50%) | 183 (41%) | 288 (53%) | 97 (67%) |  |
|  | General Practitioner | 469 (41%) | 221 (50%) | 216 (40%) | 32 (22%) |  |
|  | Specialist | 95 (8%) | 40 (9%) | 40 (7%) | 15 (10%) |  |

**Table 7: Sensitivity analysis of Table 3; numbers and percentages of patients indicating medication review and certain discussion topics about medication use as important, in total (n=3352) and stratified by levels of polypharmacy. P-values for comparisons between levels of polypharmacy.**

|  | **Total (n=3352)** | **Non-polypharmacy (n=1543)** | **Polypharmacy (n=1505)** | **Hyper-**  **polypharmacy (n=304)** | **p-value** |
| --- | --- | --- | --- | --- | --- |
| **Importance of medication review** |  | | | |  |
| Medication review | 86% | 86% | 85% | 87% | 0.125 |
| **Importance of topics about medication use** |  |  |  |  |  |
| Appropriateness of medication | 90% | 91% | 89% | 89% | 0.727 |
| Efficacy of medication | 90% | 89% | 90% | 89% | 0.998 |
| Side-effects | 86% | 86% | 85% | 86% | 0.981 |
| Risks of medication | 86% | 86% | 85% | 87% | 0.566 |
| Patients' concerns regarding medication | 69% | 68% | 69% | 71% | 0.796 |
| **Practical problems with using medication** | **51%** | **51%** | **50%** | **58%** | **0.064** |

## Appendix 5

Figure 3: Patients who had no experience with a medication review (n=3006) and reasons according to them; 3 patients did not respond. Depicted are their total responses and stratified by the level of medication use. * Significant difference across levels of polypharmacy

**Table 8: Sensitivity analysis of Table 4; numbers and percentages of topics discussed during a medication review and outcomes of the medication review, in total and stratified by the levels of polypharmacy. P-values for comparisons between levels of polypharmacy.**

| Topics discussed during medication review | Total  (n=949) | Non-polypharmacy  (n=364) | Polypharmacy  (n=465) | Hyper-polypharmacy  (n=120) |  |
| --- | --- | --- | --- | --- | --- |
|  | Agree | Agree | Agree | Agree | p-value |
| I could ask all my questions | 92% | 92% | 92% | 96% | 0.383* |
| Aim conversation was clear | 88% | 86% | 89% | 88% | 0.791* |
| Health issues were discussed | 83% | 86% | 81% | 83% | 0.267 |
| Priorities in issues | 77% | 78% | 77% | 76% | 0.641 |
| Follow-up agreements | **77%** | **79%** | **77%** | **68%** | **0.051** |
| Different treatment options | 63% | 66% | 62% | 60% | 0.391 |
| Outcomes of the medication review | (n=819) | (n=295) | (n=415) | (n=109) |  |
| Better understanding | 66% | 70% | 64% | 61% | 0.354 |
| More faith medication | 62% | 64% | 61% | 60% | 0.727 |
| Alteration in medication | 47% | 52% | 54% | 51% | 0.840 |
| Medication use improved | 49% | 52% | 48% | 45% | 0.079 |
| Fewer health issues | **38%** | **46%** | **35%** | **29%** | **<0.001** |

*Fisher’s exact test
